# Benefits of ultra-fast-track anesthesia for children with congenital heart disease undergoing cardiac surgery

**DOI:** 10.1186/s12887-019-1832-9

**Published:** 2019-12-11

**Authors:** Jing Xu, Guanghua Zhou, Yanpei Li, Na Li

**Affiliations:** Department of Anesthesiology, China Emergency General Hospital, 29 Liufangnanli Rd, Beijing, 100028 China

**Keywords:** Ultra-fast anesthesia, Congenital heart disease, Low weight children, Extubation time

## Abstract

**Background:**

To compare the outcomes of ultra-fast-track anesthesia (UFTA) and conventional anesthesia in cardiac surgery for children with congenital heart disease (CHD) and low birth weight.

**Methods:**

One hundred and ninety-four CHD children, aged 6 months to 2 years, weighting 5 to 10 kg, were selected for this study. The 94 boys and 100 girls with the American Society of Anesthesiologists (ASA) physical status III and IV were randomly divided into two groups each consisting of 97 patients, and were subjected to ultra-fast-track and conventional anesthesia for cardiac surgery. For children in UFTA group, sevoflurane was stopped when cardiopulmonary bypass (CPB) started and cis-atracurium was stopped at the beginning of rewarming, and remifentanil (0.3 μg/kg/mim) was then infused. Propofol and remifentanil were discontinued at skin closure. 10 min after surgery, extubation was performed in operating room. For children in conventional anesthesia group, anesthesia was given routinely and they were directly sent to ICU with a tracheal tube. Extubation time, ICU stay and hospital stay after operation were recorded. Sedation-agitation scores (SAS) were assessed and adverse reactions as well as other anesthesia –related events were recorded.

**Results:**

The extubation time, ICU stay and hospital stay were significantly shorter in UFTA group (*P* < 0.05) and SAS at extubation was lower in UFTA group than in conventional anesthesia group, but similar in other time points. For both groups, no airway obstruction and other serious complications occurred, and incidence of other anesthesia –related events were low.

**Conclusions:**

UFTA shortens extubation time, ICU stay and hospital stay for children with CHD and does not increase SAS and incidence of adverse reactions.

## Background

Fast-track anesthesia (FTA) is a procedure that enables extubation in intensive care unit (ICU) within 6 h after surgery to facilitate the recovery of consciousness and autonomous breathing. It has been safely applied to cardiac surgery since the 1990s [1, 2]. FTA is feasible and safe and reduces the occurrence of ventilator-induced complications, thereby decreasing ICU stay, resource use and cost [3–5]. Ultra-fast tract anesthesia (UFTA) was developed after fast-track anesthesia to further optimize the use of medical resource. With UFTA, extubation is performed immediately or within 1 h after surgery in the operating room [6]. The benefits of UFTA include lower incidence of postoperative complications, better hemodynamic performance, shorter ICU stay [7–9].

Congenital heart disease (CHD) is the most common type of congenital anomaly, occurring in up to 1% of all live births. Children with CHD often have abnormalities in brain maturation and brain injury [10, 11]. Surgery is one of the most common options for treatment [12, 13]. Anesthesia procedures are ideal for medical and cardiac surgical management. The risks of the procedures include cardiovascular and respiratory complications from anesthesia and sedation and a potentially under-appreciated risk of neurocognitive dysfunction [14] and improvement in anesthesia management would reduce the risk. In this study, we aimed to investigate the outcomes of UFTA in CHD children in cardiac surgery and to assess the effect of UFTA in reducing postoperative complication.

## Methods

### Subjects

This was a prospective study. CHD children, aged 6 months to 2 years and admitted to our hospital, were selected for this study. They weighted 5 to 10 kg with the American Society of Anesthesiologists (ASA) physical status III and IV. Children were excluded if they had respiratory tract infection within 2 weeks of surgery and organ complications. Children were also excluded if they could not interrupt ventilation during cardiopulmonary bypass (CPB) and had severe pulmonary hypertension before operation. The study protocols were approved by Ethics Committee of China Emergency General Hospital and informed consent was obtained from every guardian of child participated in the study.

### Grouping and treatment

The patients (94 boys and 100 girls) were randomly divided into two groups each consisting of 97 patients, and were subjected to UFTA and conventional anesthesia before surgeries. The surgeries were performed by the same team of surgeons, anesthesiologists, and post-operative physicians. For children in UFTA group, cis-atracurium was stopped and remifentanil (0.3 μg/kg/min) was infused at rewarming. At the onset of skin closure, propofol and remifentanil were discontinued. During operation, the patients were continuously infused with dexmedetomidine (1 μg/kg/h) till they were moved to ICU. After operation, 0.375% ropivacaine was used to block the peripheral nerves. Patients were switched to spontaneous ventilation after a trial of spontaneous ventilation using synchronous intermittent mandatory ventilation (SIMV). Extubation was performed within 10 min after surgery in the operating room and the patients were sent to ICU with facemask to supply oxygen (target SpO2 94–100%). Children in conventional anesthesia group were supplemented with midazolam (0.05 mg/kg) and sufentanil (1 μg/kg) at rewarming after CPB. After surgery, the anesthetics were stopped and the patients were sent to ICU with a tracheal tube. They were extubated once all signs were normal as reported [15].

### Anesthesia method and hemodynamic monitoring

No preoperative medication was used in all children. ECG, HR, RR and SPO_2_ were monitored in the operating room. Anesthesia was induced using ketamine (1–2 mg/kg), atropine (0.01 mg/kg), midazolam (0.05–0.10 mg/kg), cis-atracurium (0.1–0.2 mg/kg) and sufentanil (0.5–1.0 μg/kg). Mechanical ventilation was given via orotracheal intubation. Tidal volume (VT) was set at 10 ml/kg, Fraction of inspiration O_2_ (FiO_2_) at 40–50%, respiratory-exchange ratio (RR) at 22–24 time/min, inspiration (I): expiration (E) at 1:2, end-tidal partial pressure of carbon dioxide (PETCO_2_) at 35–40 mmHg. After the induction, blood pressure (BP) was monitored via a radial artery catheter and central venous pressure (CVP) was monitored via catheter placed in right internal jugular vein. Anesthesia was maintained through the inhalation of 1–2% sevoflurane, and the patients were infused with propofol (3 mg/kg/h), cis-atracurium (0.1 mg/kg/h) and dexmedetomidine (1 μg/kg/h) throughout the surgery. The concentration of sevoflurane was adjusted based on hemodynamics. Additional midazolam (0.05 mg/kg) and sufentanil (1 μg/kg) were given before skin incision. Sevoflurane was discontinued when CPB started.

Postoperative pain was assessed using visual analogue scale (VAS) as reported [16], and morphine (10 μg/kg/h) was infused if VAS was > 4 and stopped if VAS was < 2. The Riker Sedation–Agitation Scale (SAS) was used to assess the sedation-agitation status after surgery [17], and dexmedetomidine (0.2 μg/kg/h) was infused if SAS was > 5.

### Assessment

MAP, HR and CVP were recorded before anesthesia induction (T_0_), after intubation (T_1_), at incision (T_2_), before and during CPB (T_3_ and T_4_), before and after extubation (T_5_ and T_6_). Extubation time (the interval between the end of operation and extubation), ICU stay and post-operative hospitalization stay were also recorded. SAS at extubation, and 6, 12 and 24 h after operation were assessed. Adverse reactions (airway obstruction) as well as other relevant events after operation were recorded.

### Statistical analysis

The data were analysed by SPSS version 20.0 for Windows (SPSS Inc., Chicago, IL, USA). The normality of distribution of continuous variables was tested by one-sample Kolmogorov-Smirnov test. Continuous variables with normal distribution were presented as mean ± s.d. (standard derivation); non-normal variables were reported as median (interquartile range [IQR]). Means of 2 continuous normally distributed variables were compared by independent samples Student’s t test. The frequencies of categorical variables were compared using Pearson χ^2^ or Fisher’s exact test, when appropriate. A value of *P* < 0.05 was considered significant.

## Results

A total of 194 children were enrolled in this study, 97 in each group. Two groups of patients had no difference in gender, age, body weight, CHA classification, ASA grade, surgical methods, anesthesia time, CPB time and block time (Table 1).

**Table 1:** Baseline comparison of children underwent ultra-fast track anesthesia and conventional anesthesia

Parameters	Ultra-fast track anesthesia (*n* = 97)	Conventional anesthesia (*n* = 97)	*P* value
Male/female (no.)	45/52	49/48	0.231
Age (year)	1.2 ± 0.5	1.1 ± 0.5	0.331
Body weight (kg)	9.1 ± 1.1	9.2 ± 1.1	0.289
No. pre-term patients	46	44	0.782
Birth weight (kg)	2.3 ± 0.46	2.2 ± 0.38	0.389
ASAIII /VI (no.)	52 / 45	46 / 51	0.254
CHD classification			
Atrial septal defect (no.)	48	52	0.612
Ventricular septal defect (no.)	30	30	0.452
Atrioventricular septal defect	2	3	0.652
D-transposition of the great arteries	4	3	0.978
Tetralogy of fallot (no.)	19	15	0.334
Coarctation of the aorta	2	3	0.778
Interruption arterial arch	1	1	0.978
Pulmonary stenosis	5	4	0.878
Ventricular outflow tract obstruction	6	8	0.478
Anesthesia time (h)	3.4 ± 1.1	3.3 ± 1.0	0.342
Surgery time (min)	297.1 ± 22.8	289.0 ± 20.5	0.551
CPB time (min)	47.4 ± 11.8	46.3 ± 10.7	0.234
Block time (min)	30.2 ± 8.9	31.4 ± 9.1	0.331

Furthermore, no difference in MAP, HR and CVP was observed between the two groups at different time points (Table 2). However, extubation time, ICU stay and hospital stay were significantly shorter in the UFTA group than in conventional group (*P* < 0.05, Table 3).

**Table 2:** Comparison of MAP, HR and CVP between children undergoing ultra-fast track anesthesia and conventional anesthesia

Parameters	Anesthesia	No. case	T_0_	T_1_	T_2_	T_3_	T_4_	T_5_	T_6_
MAP	UTFA	97	60.9 ± 5.6	56.9 ± 4.2	56.6 ± 4.1	50.2 ± 5.4	30.1 ± 2.2	59.4 ± 3.9	59.1 ± 3.6
(mmHg)	Conventional	97	60.5 ± 5.3	57.3 ± 4.0	56.4 ± 4.5	49.6 ± 4.8	29.5 ± 2.4	61.4 ± 4.2	60.2 ± 4.8
HR	UTFA	97	130.4 ± 4.3	129.3 ± 4.3	124.4 ± 4.9	128.4 ± 4.3	/	136.4 ± 4.6	136.4 ± 6.3
(time/m)	Conventional	97	129.4 ± 4.1	130.4 ± 4.1	131.4 ± 4.3	131.4 ± 4.3	/	137.2 ± 4.8	136.8 ± 5.8
CVP	UTFA	97	4.5 ± 0.9	4.9 ± 0.7	5.3 ± 1.0	5.3 ± 1.1	/	6.4 ± 0.3	6.4 ± 0.4
(mmHg)	Conventional	97	4.6 ± 0.8	5.0 ± 0.9	5.2 ± 1.2	5.2 ± 1.0	/	6.8 ± 0.5	6.8 ± 0.4

**Table 3:** Comparison of extubation time, ICU stay, postoperative hospital stay and SAS scores between children undergoing ultra-fast track anesthesia and conventional anesthesia

	Ultra-fast track anesthesia	Conventional anesthesia
No. case	97	97
Extubation time (min)	22.9 ± 3.5^a^	189.1 ± 31.2
ICU stay (h)	20.7 ± 6.5^a^	28.5 ± 4.2
Postoperative hospital stay (d)	11.5 ± 3.0^a^	16.1 ± 2.4
SAS scores		
At extubation	3.8 ± 0.6^a^	4.8 ± 0.7
6 h- postoperation	3.9 ± 0.4	3.9 ± 0.6
12 h- postoperation	4.0 ± 0.6	4.0 ± 0.6
24 h- postoperation	4.0 ± 0.5	3.9 ± 0.5

^a^*P* < 0.05 vs conventional anesthesia

The SAS score of UFTA group was significantly lower than that of traditional anesthesia group at extubation (*P* < 0.05), but the scores were similar 6, 12 and 24 h after operation (Table 3). Other anesthesia-related parameters such as the incidence of continuous positive airway pressure (CPAP) use and reintubation rate were similar between the two groups, but the number of patients with ventilator-associated pneumonia was less in UFTA group than in conventional group (*P* < 0.05, Table 4), although the numbers were small in both groups. The incidence of adverse events were low and similar in both group and no airway obstruction was not observed in either group (Table 5).

**Table 4:** Comparison of ventilator-associated pneumonia and continuous positive airway pressure use and reintubation rate between children undergoing ultra-fast track anesthesia and conventional anesthesia

	Ultra-fast track anesthesia	Conventional anesthesia
No. case	97	97
Ventilator-associated pneumonia (n)	3	5^a^
Continuous positive airway pressure use (n)	3	3
Reintubation (n)	5	6
Respiratory tract infections (n)	3	4

^a^*P* < 0.05 vs conventional anesthesia

**Table 5:** Comparison of adverse events between children undergoing ultra-fast track anesthesia and conventional anesthesia

Adverse event	Ultra-fast track anesthesia	Conventional anesthesia
No. case	97	97
Airway obstruction	0	0
Arrhythmia	1	1
Infection	1	2
Bleeding	1	1
Pneumothorax	0	0

^a^*P* < 0.05 vs conventional anesthesia

## Discussion

Our results show that the extubation time is significantly shorter in the UFTA group than in conventional group. Furthermore, the ICU stay and hospitalization stay are also shorter. No serious hemodynamic changes, nor serious complications are observed in neither groups, confirming that UFTA is safe for anesthesia management in CHD operation.

UFTA was developed to optimize perioperative anesthesia operations and management to shorten intubation time after operation for fast recovery of patients. A Meta-analysis of randomized controlled trials with large sample size showed that compared with conventional anesthesia management, UFTA is relatively low-risk and safe in terms of fatality and mortality with shorter extubation time and ICU stay [18].

Prolonged tracheal intubation and mechanical ventilation are major risk factors for respiratory-related complications [19]. A large number of studies have shown that compared with conventional anesthesia management for cardiac surgery, extubation in the operating room after surgery reduces the use of muscle relaxants, facilitates the restoration of spontaneous breathing, decreases the risks of ventilator-related iatrogenic lung inflammation, respiratory tract damage and other pulmonary complications [20]. A propensity score matching analysis showed that the use of UFTA in patients with low to moderate risks of cardiac surgery would improve cost-effectiveness and outcomes as compared to conventional anesthesia management [21]. A prospective observational study showed that extubation in the operating room was successful in 87.1% of the patients without any increase in mortality and morbidity, but with a decrease in ICU length of stay and less use of hospital resources [22].

For CHD surgery, the optimization in UFTA mainly includes perioperative anesthesia managements, such as anesthesia method, selection of anaesthetics, control of perioperative body temperature and postoperative analgesia. In the present study, all children were given a combined intravenous-inhalational anesthesia with sufentanil before CPB. The anesthetic depth was adjusted based on the circulation to reduce the stress induced by extubation and thoracotomy. Remifentanil and propofol infused through the veins after postoperative rewarming in the UFTA group, which was used to provide sedative and analgesic effect and minimize surgical stimulation-induced stress and intraoperative awareness, are ultra-short-acting. They also reduce the dose of sufentanil during operation for better early extubation and postoperative respiratory depression and duration of ventilation time. Studies have also shown that reducing the use of narcotics and analgesics help the recovery of pulmonary function and gastrointestinal function [21].

Perioperative body temperature is a major factor affecting extraction after cardiac surgery [23]. In the present study, body temperature was kept above 36.0 °C. This would accelerate the metabolism of anesthetics and muscle relaxants for better homeostasis of internal environment. Postoperative analgesia can affect extubation and prognosis after cardiac surgery. We used ropivacaine and dexmedetomidine combined with morphine for analgesia in UTFA group. The outcomes are satisfactory and no adverse events such as post-operative agitation were observed. This is important for better and early recovery of pulmonary function.

There are also limitations in this study. For example, hematological parameters related to ventilator-associated pneumonia, such as procalcitonin was not measured; the size of sample was relatively small and most of the patients had mild illness without severe pulmonary artery hypertension before operation. Therefore, studies with larger sample size and more complicated CHD surgeries are needed to further validate the feasibility of UFTA in CHD children.

## Conclusions

UFTA generates stable hemodynamics during operation, shorter extubation time, shorter ICU and hospitalization stay without increase in adverse reactions. It is worthy of recommendation for clinical practice.

## Data Availability

The datasets used and/or analysed during the current study are available from the corresponding author on reasonable request.

## Abbreviations

ASA: American Society of Anesthesiologists
BP: Blood pressure
CHD: Congenital heart disease
CPB: Cardiopulmonary bypass
CVP: Central venous pressure
E: Expiration
ICU: Intensive care unit
PETCO_2_: Partial pressure of carbon dioxide
RR: Respiratory-exchange ratio
SAS: Sedation-agitation scores
SIMV: Synchronous intermittent mandatory ventilation
UFTA: Ultra-fast-track anesthesia
VAS: Visual analogue scale
VT: Tidal volume
